# Role of drug transporters and drug accumulation in the temporal acquisition of drug resistance

**DOI:** 10.1186/1471-2407-8-318

**Published:** 2008-11-03

**Authors:** Stacey L Hembruff, Monique L Laberge, David J Villeneuve, Baoqing Guo, Zachary Veitch, Melanie Cecchetto, Amadeo M Parissenti

**Affiliations:** 1 Regional Cancer Program, Sudbury Regional Hospital, Sudbury, ON Canada; 2 Department of Chemistry and Biochemistry, Laurentian University, Sudbury, ON Canada; 3 Department of Biology, Laurentian University, Sudbury, ON Canada; 4 Division of Medical Sciences, Northern Ontario School of Medicine, Sudbury, ON Canada

## Abstract

**Background:**

Anthracyclines and taxanes are commonly used in the treatment of breast cancer. However, tumor resistance to these drugs often develops, possibly due to overexpression of drug transporters. It remains unclear whether drug resistance *in vitro *occurs at clinically relevant doses of chemotherapy drugs and whether both the onset and magnitude of drug resistance can be temporally and causally correlated with the enhanced expression and activity of specific drug transporters. To address these issues, MCF-7 cells were selected for survival in increasing concentrations of doxorubicin (MCF-7_DOX-2_), epirubicin (MCF-7_EPI_), paclitaxel (MCF-7_TAX-2_), or docetaxel (MCF-7_TXT_). During selection cells were assessed for drug sensitivity, drug uptake, and the expression of various drug transporters.

**Results:**

In all cases, resistance was only achieved when selection reached a specific threshold dose, which was well within the clinical range. A reduction in drug uptake was temporally correlated with the acquisition of drug resistance for all cell lines, but further increases in drug resistance at doses above threshold were unrelated to changes in cellular drug uptake. Elevated expression of one or more drug transporters was seen at or above the threshold dose, but the identity, number, and temporal pattern of drug transporter induction varied with the drug used as selection agent. The pan drug transporter inhibitor cyclosporin A was able to partially or completely restore drug accumulation in the drug-resistant cell lines, but had only partial to no effect on drug sensitivity. The inability of cyclosporin A to restore drug sensitivity suggests the presence of additional mechanisms of drug resistance.

**Conclusion:**

This study indicates that drug resistance is achieved in breast tumour cells only upon exposure to concentrations of drug at or above a specific selection dose. While changes in drug accumulation and the expression of drug transporters does occur at the threshold dose, the magnitude of resistance cannot be attributed solely to changes in drug accumulation or the activity of drug transporters. The identities of these additional drug-transporter-independent mechanisms are discussed, including their likely clinical relevance.

## Background

While anthracyclines and taxanes are highly effective drugs used in the treatment of breast and other cancers, tumour drug resistance mechanisms limit their clinical effectiveness. Tumours can either be intrinsically resistant to chemotherapy agents, or acquire resistance upon exposure to a previous round of chemotherapy [1]. Drug resistance, whether intrinsic or acquired, is believed to cause treatment failure in over 90% of patients with metastatic cancer [2]. Thus, it is critical that clinically relevant mechanisms for drug resistance be elucidated in order to identify approaches to circumvent drug resistance. A wide variety of proteins or protein mutations have been found to play a role in drug resistance *in vitro*, but their relevance clinically remains to be established [2-4].

To date, six drug transporters have been shown to play a role in multidrug resistance in tumour cells *in vitro*. These include ABCB1 (P-glycoprotein), ABCC1 (MRP1), ABCC2 (MRP2), ABCC4 (MRP4), ABCG2 (BCRP), and the lung resistance protein (LRP). Of these, three are overexpressed in the large majority of tumour cell lines that have been successfully selected for resistance to anthracyclines and taxanes. These include ABCB1, ABCC1, and ABCG2, and all play a role in reducing the ability of tumour cells to accumulate specific chemotherapy drugs [5,6]. Although these transporters are unique in their sequences, there is some overlap in the drugs that can be transported by each protein. ABCC1 confers resistance to doxorubicin, daunorubicin, vincristine, etoposide, epirubicin, chlorambucil, methotrexate, melphalan and paclitaxel [5,7-9]. ABCC2 has been shown to be associated with resistance to doxorubicin, etoposide, methotrexate, irinotecan (SN-38), vincristine, vinblastine, camptothecin (CPT-11) [9], paclitaxel, docetaxel, etoposide, mitoxantrone [10] and cisplatin [11]. ABCC4 is believed to confer resistance to the camptothecins (SN-38, rubitecan, irinotecan), cyclophosphamide [12], topotecan [13], methotrexate, and nucleoside analogues [14]. Numerous studies have been conducted on ABCB1 and its ability to transport chemotherapy drugs. It has been shown to directly transport vinblastine, paclitaxel, docetaxel, daunorubicin, doxorubicin, epirubicin, etoposide, teniposide, and mitoxantrone [9,15-18]. The final ABC transporter (ABCG2) confers resistance to mitoxantrone, doxorubicin, epirubicin, daunorubicin, vinca alkaloids, paclitaxel, cisplatin, etoposide, teniposide, irinotecan, topotecan, and camptothecin [9,19-24]. Although not an ABC transporter, lung resistance-related protein (LRP) is a human major vault protein whose expression appears to correlate with resistance to doxorubicin, mitoxantrone, methotrexate, etoposide, vincristine, and cisplatin [reviewed in [25]]. While the exact cellular function of the major vault proteins (MVP) remains to be elucidated, the majority of these proteins have been shown to interact with cytoskeletal elements or within the nucleus—in particular nucleoli, the nuclear membrane and/or the nuclear pore complex [25-27]. Elevated levels of MVPs have been observed in some drug-resistant cell lines. While there is little direct evidence that the proteins can directly transport chemotherapy drugs, it has been shown that overexpression of LRP alters the subcellular distribution of doxorubicin, such that the drug localizes to cytoplasmic organelles rather than to DNA within the nucleus [28].

Despite the overwhelming evidence that drug transporters can confer resistance to a variety of chemotherapy agents in tumour cells *in vitro*, attempts to use their expression as definitive biomarkers for the identification of drug resistant tumours have met with mixed success [29-32]. In addition, administration of drug transporter inhibitors (in particular for ABCB1) to prevent or reverse drug resistance in cancer patients has largely been unsuccessful, in part due to the toxicity of these compounds [33,34]. Given these findings, it is likely that additional mechanisms may play an equal or much greater role in clinical resistance to chemotherapy drugs. Inhibition of these targets may prove more fruitful in combating drug resistance in patients. To rigorously assess the temporal and causal relationships between the acquisition of drug resistance and the induction of drug transporters and drug accumulation defects *in vitro*, we selected MCF-7 breast tumour cells for survival in increasing concentrations of paclitaxel, docetaxel, doxorubicin, or epirubicin. We then examined cells during selection for their expression of various drug transporters, their sensitivity to various chemotherapy agents, their ability to uptake drugs, and their sensitivity to a pan-ABC drug transporter inhibitor. Our findings suggest that changes in cellular drug accumulation do temporally correlate with the acquisition of drug resistance at clinically relevant drug doses. However, the onset of drug resistance is not always correlated with the induction of specific drug transporters. Moreover, inhibition of drug transporter function and/or restoration of drug accumulation has only limited to no ability to restore sensitivity to chemotherapy agents. Additional mechanisms which are temporally and functionally correlated with the acquisition of drug resistance are discussed.

## Methods

### Selection of MCF-7 Cells for Resistance to Various Chemotherapy Drugs

MCF-7 cells were selected for progressive resistance to doxorubicin or paclitaxel as previously described [35] except that an aliquot of cells was stored before each escalation in drug dose. Selection began at a drug dose (dose 1) that was 1000-fold less than the concentration at which 50% of parental MCF-7 cells are killed (the IC_50_). The dose was then increased 1.5- or 3-fold until the maximally tolerated dose was achieved. Similar selections were performed in an identical manner to obtain cells exhibiting progressive resistance to epirubicin or docetaxel. Table 1 depicts the drug concentrations (doses) to which the cells were exposed. The panel of cell lines selected for progressive resistance to paclitaxel, docetaxel, doxorubicin, or epirubicin were referred to as MCF-7_TAX-2_, MCF-7_TXT_, MCF-7_DOX-2_, and MCF-7_EPI _cells, respectively, and the dose to which cells were selected noted. For example, MCF-7_DOX-2_cells selected to dose 9 refers to MCF-7 cells that were exposed to step-wise increases in the concentration of doxorubicin until the selection dose reached 29.1 nM doxorubicin (see Table 1). MCF-7_TAX-2 _and MCF-7_DOX-2 _were given the "-2" nomenclature to distinguish these cells from paclitaxel- and doxorubicin-resistant cell lines previously isolated by our laboratory (MCF-7_TAX _and MCF-7_DOX_, respectively) [35-37]. During each selection for drug resistance, an identical "selection" was performed in the absence of drug ("co-cultured control" or MCF-7_CC _cells) in order to account for any changes in drug sensitivity or other cell phenotypes associated with long-term propagation in culture.

**Table 1:** Concentrations of chemotherapy drugs used at each selection dose

**Dose**	**Doxorubicin**	**Epirubicin**	**Paclitaxel**	**Docetaxel**
IC_50_	8.91 nM	4.79 nM	0.56 nM	0.51 nM
1	8.91 pM	4.79 pM	0.56 pM	0.51 pM
2	26.7 pM	14.4 pM	1.67 pM	1.52 pM
3	80.1 pM	43.2 pM	5.01 pM	4.56 pM
4	240 pM	130 pM	15.0 pM	13.7 pM
5	720 pM	390 pM	45.0 pM	41.1 pM
6	2.16 nM	1.17 nM	135 pM	123 pM
7	6.48 nM	3.51 nM	405 pM	369 pM
8	19.4 nM	10.5 nM	1.22 nM	1.11 nM
9	29.1 nM	31.5 nM	3.66 nM	3.33 nM
10	43.6 nM	94.5 nM	11.0 nM	5.00 nM
11	65.4 nM	284 nM	33.0 nM	15.0 nM
12	98.1 nM*	852 nM	99.0 nM	45.0 nM*

MCF-7 breast tumour cells were exposed to progressively higher concentrations (doses) of chemotherapy drugs, beginning at a dose equivalent to 1/1000^th ^of the concentration required to kill or inhibit the growth of 50% of wild-type MCF-7 cells (the IC_50_). The symbol * denotes the highest concentration of drug at which the cells could survive (the maximally tolerated dose).

### Measurement of Cellular Drug Sensitivity

Drug sensitivity for cells at various selection doses was measured using a clonogenic assay as described previously [35]. The effect of 5 μM cyclosporin A on drug sensitivity was also determined using a clonogenic assay, except that only cells selected to doses 9 or 12 were used and the agent was added 1 hour prior to the addition of chemotherapy drugs. The concentration at which 50% of cells are killed (the IC_50 _value) was computed for both MCF-7_CC _cells and cells selected for resistance at specific selection doses. Resistance factors for cells at each selection dose were then computed by dividing the IC_50 _value for the drug-selected cells by the IC_50 _value for MCF-7_CC _cells at that dose.

### Measurement of 3H-Paclitaxel Uptake Into Cells

Radiolabelled paclitaxel uptake into cells was monitored as described previously [35], except that some cells were pre-treated with 5 μM cyclosporin A rather than 2 μM valspodar. All values presented were an average of 3 trials, and the percent uptake expressed relative to uptake into MCF-7_CC _cells at 24 hours.

### Measurement of Doxorubicin and Epirubicin Uptake Into Cells

Doxorubicin uptake into cells was monitored as described previously [35] with the exception that 4.0 × 10^5 ^cells were used in the assay, and pre-treatment of some cells was with 5 μM cyclosporin A rather than 2 μM valspodar. All values presented were an average of 3 trials, and the percent uptake expressed relative to uptake into MCF-7_CC _cells at 16 hours. Epirubicin uptake was performed in an identical manner to that of doxorubicin uptake.

### Quantification of Drug Transporter Transcripts

Quantification of cellular transcripts for various drug transporters was conducted by "real time" quantitative reverse transcription PCR (Q-PCR) using RNA isolated from cells at various selection doses and gene-specific primers as described previously [38]. The primers used were: S28: 5'-TCCATCATCCGCAATGTAAAAG-3' and 5'-GCTTCTCGCTCTGACTCCAAA-3'; ABCB1: 5'-GCAGCTGGAAGACAAATACACAA-3' and 5'-CCCAACATCGTGCACA- TCA-3'; ABCC1: 5'-GCTGGAGTGTGTGGGCAACT-3' and 5'-CTGAGGCTGTGCCT-GGAGAT-3'; ABCC2: 5'-TCCTTGCGCAGCTGGATTACAT-3' and 5'-TCGCTGAAGTGA-GAGTAGATTG-3'; ABCC4: 5'-CCCCTGAAGGCTTCTTGTTAGA-3' and 5'-GGGTAC-ACACTCCCTACTACAATGTC-3'; ABCG2: 5'AACCTGGTCTCAACGCCATC-3' and 5'-GTCGCGGTGCTCCATTTATC-3'; LRP/MVP: 5'-CAGCTGGCCATCGAGATCA-3' and 5'-CATCCCGAGACACAGGGTTG-3'.

### cDNA Microarray Analysis of Wildtype and Drug-Resistant Cell Lines

cDNA microarray analyses were performed using RNA isolated from MCF-7_CC_, MCF-7_DOX-2_, MCF-7_EPI_, MCF-7_TAX-2_, and MCF-7_TXT _cells (selected to dose level 12) as described previously [37]. Human microarrays (1.7v8) were from the University Health Network (Toronto, ON). These arrays were used to assess the level of expression of over 1700 unique gene sequences. After scanning, the microarrays were analysed and Significance Analysis of Microarrays (SAM) graphs generated using the Multiexperiment Viewer v4 software, which is part of the TM4 software suite available freely from the J. Craig Venter Institute .

### Immunoprecipitation and Immunoblotting of ABCB1 and ABCC1

Protein extracts from MCF-7_CC_, MCF-7_DOX-2_, MCF-7_EPI_, MCF-7_TAX-2_, and MCF-7_TXT _cells (selected to dose level 12) were prepared by washing adherent cells in PBS and scraping the cells in a lysis buffer containing 20 mM Tris pH 7.5, 150 mM NaCl, 1 mM EDTA, 1 mM EGTA, 1% Triton X-100, and 1 Complete™ Protease Inhibitor Tablet for every 50 ml of prepared buffer. After homogenization and clarification of the lysate, 300 μg of total protein from each lysate was incubated with 5 μl of either an ABCB1-specific antibody (C219 – Cedarlane Laboratories, Burlington, ON) or ABCC1-specific antibody (QCRL-1, Santa Cruz Biotechnology Inc, Santa Cruz, CA) for 1 hour on ice. After the incubation with the antibody, 25 μl of protein A/G sepharose was added and allowed to incubate overnight at 4°C on a rocker. The following day, the tubes were centrifuged for 5 minutes at 16000 × g and the resulting pellet was washed 4 times with lysis buffer. The sepharose pellet was then resuspended in equal volumes of lysis buffer and gel loading buffer, and the entire sample was loaded onto an 8% SDS-polyacrylamide gel. Western blotting experiments were then performed using standard procedures. Membranes, after blocking, were incubated overnight at 4°C with a 1:100 dilution of either primary antibody, followed by the appropriate secondary antibody for 1 hour at room temperature.

### Statistical Analyses

To determine if differences in paclitaxel, doxorubicin and epirubicin uptake were statistically significant, a two factor ANOVA (with replication) was performed with a Tukey's post hoc test. To determine if differences in gene levels seen in the four different cell lines using Q-PCR were statistically significant, the non-parametric Kruskal-Wallis test was used.

## Results

### Assessment of Isogeneity Between MCF-7CC Cells and Its Drug-Resistant Variants

**Figure 1:**
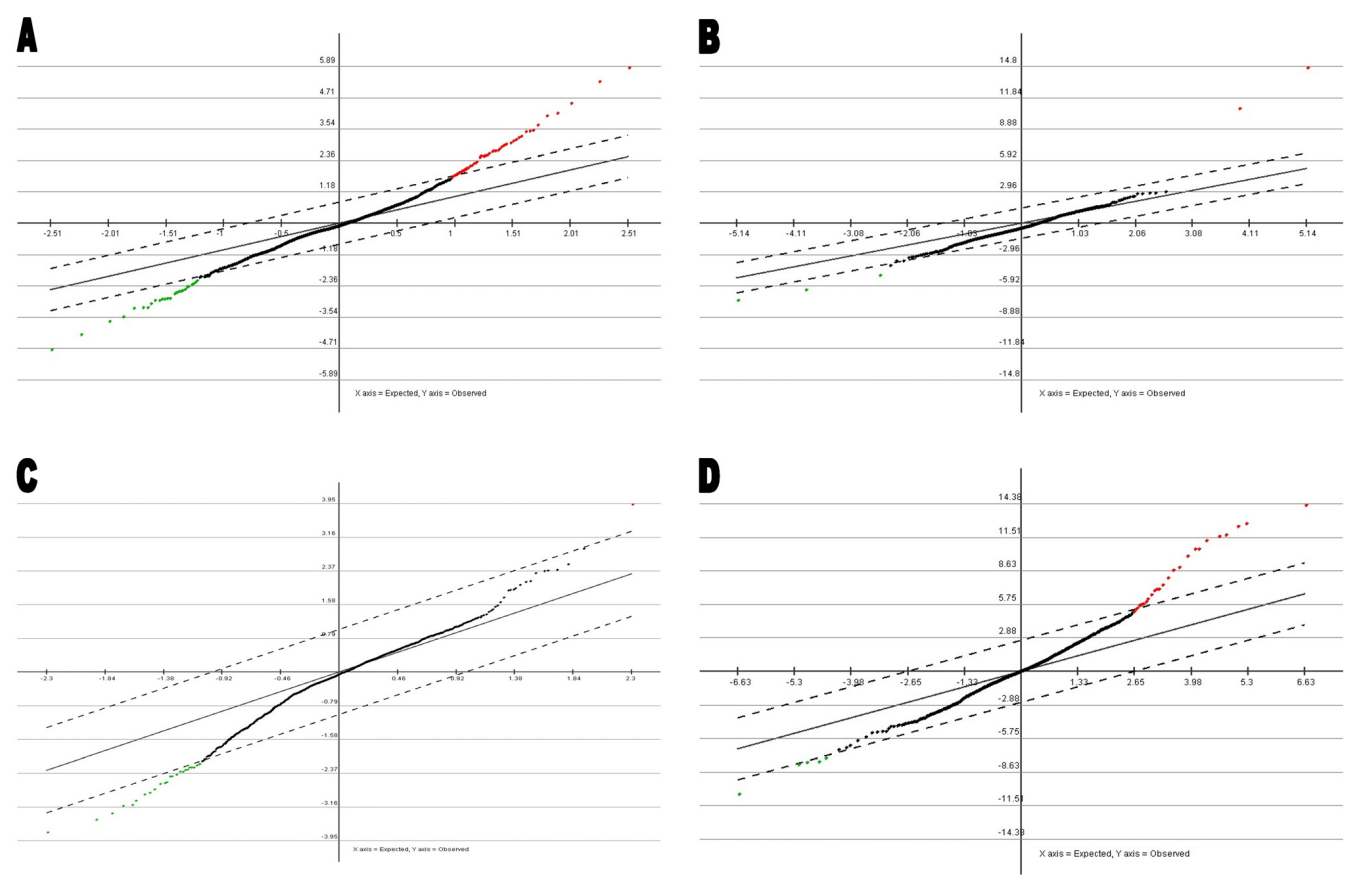
**Significance Analysis of Microarrays (SAM) graphs depicting similarities and differences in gene expression between the MCF-7_DOX-2_(A), MCF-7_EPI _(B), MCF-7_TAX-2 _(C), or MCF-7_TXT _(D) cell lines and their co-cultured parental (MCF-7_CC_) cell line. **Genes not found to be different in expression from MCF-7_CC _cells are depicted in black, while upregulated and downregulated genes are depicted as red and green dots, respectively.

cDNA microarray analyses were performed on RNA extracted from MCF-7_CC_, MCF-7_DOX-2_, MCF-7_EPI_, MCF-7_TAX-2_, and MCF-7_TXT _cells (selected to dose level 12). The SAM plots from such analyses (Figure 1) indicated that the expression levels of the vast majority of genes in MCF-7_DOX-2_, MCF-7_EPI_, MCF-7_TAX-2_, and MCF-7_TXT _cells were very similar to MCF-7_CC _cells. This strongly suggests that the cell lines are isogenic and that the drug-resistant derivative cell lines do not stem from rare variants or contaminating cells within the population. A number of genes were upregulated or downregulated in the drug-resistant cell lines relative to MCF-7_CC _cells (red and green spots on SAM plots, respectively), but these were few in number and likely relate to changes in gene expression associated with the acquisition of drug resistance. MCF-7_DOX-2 _cells exhibited the greatest changes in gene expression, while MCF-7_EPI _cells showed the fewest changes. The microarray data thus suggest that a variety of genes and their protein products contribute to acquired drug resistance in breast tumour cells.

### Tolerance of MCF-7 Cells to Increasing Concentrations of Anthracyclines and Taxanes

Selection of cells in the presence of increasing concentrations of doxorubicin, epirubicin, paclitaxel, or docetaxel (MCF-7_DOX-2_, MCF-7_EPI_, MCF-7_TAX-2 _and MCF-7_TXT _cells, respectively) was carried out simultaneously under identical culture conditions (see Materials and Methods). Beginning at a concentration 1000-fold less than the empirically-derived IC_50 _for each drug (dose 1), MCF-7_TAX-2 _and MCF-7_EPI _cells survived 3-fold increases in drug dose until the maximally tolerated dose was reached or selection was stopped. In contrast, MCF-7_DOX-2 _cells survived only 1.5-fold increases in the doxorubicin selection dose at or above dose 9 until the maximally tolerated dose (98.1 nM) was reached (dose 12). MCF-7_TXT _cells were unable to survive a 3-fold increase at dose 10, but were able to survive subsequent 3-fold increases in the selection dose until the maximally tolerated dose (45 nM) was reached (dose 12).

### A Threshold Drug Concentration is Required for Acquisition of Resistance to Anthracyclines and Taxanes

Clonogenic assays were conducted to measure the sensitivity of cells to doxorubicin, epirubicin, paclitaxel, or docetaxel during selection in increasing concentrations of each of the 4 chemotherapy drugs. The IC_50 _values for each drug in each of the cell lines (including MCF-7_CC _cells) at the various selection doses were then determined and resistance factors computed as described in Materials and Methods. No resistance to the 4 chemotherapy agents was seen in any of the cell lines when the selection dose was less than or equal to dose 7 (data not shown). Even at dose 8 (which is closest to the IC_50 _value of MCF-7 cells for each drug), little to no drug resistance was observed (Table 2). However, when dose 9 was reached, resistance to the selection agent and to a drug of similar structure were very apparent (Table 2). Interestingly, this suggests that the drug used during selection must reach a specific threshold concentration before any degree of drug resistance is achieved. As shown in Table 2, this threshold dose was typically dose 9 (which exceeds the IC_50 _for MCF-7 cells by between 3.3- and 6.6-fold).

**Table 2:** Resistance factors and relative drug uptake for MCF-7 breast tumour cells selected for survival in increasing concentrations (doses) of chemotherapy drugs

**ll Line**	**Treatment**	**Dose 8**		**Dose 9**		**Dose 10**		**Dose 11**		**Dose 12**	
		***Resistance Factor***	***Relative Uptake***	***Resistance Factor***	***Relative Uptake***	***Resistance Factor***	***Relative Uptake***	***Resistance Factor***	***Relative Uptake***	***Resistance Factor***	***Relative Uptake***

MCF-7_DOX-2_	Doxorubicin	1.80	93%	2.48	46%*	3.59	37%**	42.5	37%**	27.8	33%***
	Epirubicin	0.84	95%	2.91	38%**	6.99	29%***	14.8	27%*	4.79	28%**
MCF-7_EPI_	Doxorubicin	1.82	108%	39.2	17%***	130.8	13%***	391.6	13%***	203.4	19%***
	Epirubicin	1.05	114%	93.9	11%***	422.6	9%***	486.2	9%**	815.3	14%**
MCF-7_TAX-2_	Paclitaxel	1.00	115%	19.9	16%***	119.2	10%***	156.3	3%***	535.2	2%***
	Docetaxel	0.67		8.19		25.4		37.5		72.6	
MCF-7_TXT_	Paclitaxel	1.35	77%	29.4	30%***	16.9	17%***	148	28%**	251	9%***
	Docetaxel	1.05		15.9		3.00		30.8		79.2	

MCF-7 cells exhibiting progressive resistance to doxorubicin, epirubicin, paclitaxel, or docetaxel were obtained (MCF-7_DOX-2_, MCF-7_EPI_, MCF-7_TAX-2_, and MCF-7_TXT _cells, respectively). Drug sensitivity for each drug-resistant cell line and MCF-7_CC _cells at that selection dose was measured using a clonogenic assay. Resistance factors were then calculated by dividing the IC_50 _value for the drug-resistant cell lines by the IC_50 _value for MCF-7_CC _cells. Drug uptake into drug-resistant cell lines was also measured as described in Materials and Methods and expressed relative to uptake into MCF-7_CC _cells. Statistical analyses were conducted to determine if the drug uptake in the resistant cells varied significantly from MCF-7_CC _cells (* p < 0.05, ** p < 0.01 and *** p < 0.001).

MCF-7_DOX-2 _cells selected to dose 9 exhibited a 2.5-fold resistance to doxorubicin and a 2.9-fold cross resistance to epirubicin. Resistance factors increased as the selection dose increased, resulting in a 28-fold resistance to doxorubicin and a 4.8-fold cross-resistance to epirubicin for MCF-7_DOX-2 _cells at dose 12. In contrast to the doxorubicin-resistant cells, MCF-7_EPI _cells showed larger resistances at dose 9 (94-fold resistance to epirubicin and 39-fold resistance to doxorubicin). These resistances increased with increasing selection dose, culminating with 203-fold resistance to doxorubicin and 815-fold resistance to epirubicin at dose 12.

MCF-7 cells exposed to increasing concentrations of taxanes also developed resistance to these agents beginning with dose 9 and increasing with selection dose. MCF-7_TAX-2 _cells were 19.9-fold resistant to paclitaxel and 8.19-fold cross-resistant to docetaxel at dose 9, increasing to 535-fold and 72.6-fold resistance to paclitaxel and docetaxel, respectively, at dose 12. Interestingly, cells selected for resistance to docetaxel acquired cross resistance at dose 9 to paclitaxel (29.4-fold), which exceeded resistance to the selection agent (15.9-fold). While resistance increased with higher selection doses, the magnitude of cross-resistance in MCF-7_TXT _cells to paclitaxel at dose 12 (251-fold) was still greater than resistance to docetaxel (79.2-fold). While anthracycline and taxane resistance generally increased with increasing selection dose, the magnitude of the resistance factor at each selection dose varied significantly from experiment to experiment.

### Relationship between Drug Resistance and Cellular Paclitaxel Uptake

**Figure 2:**
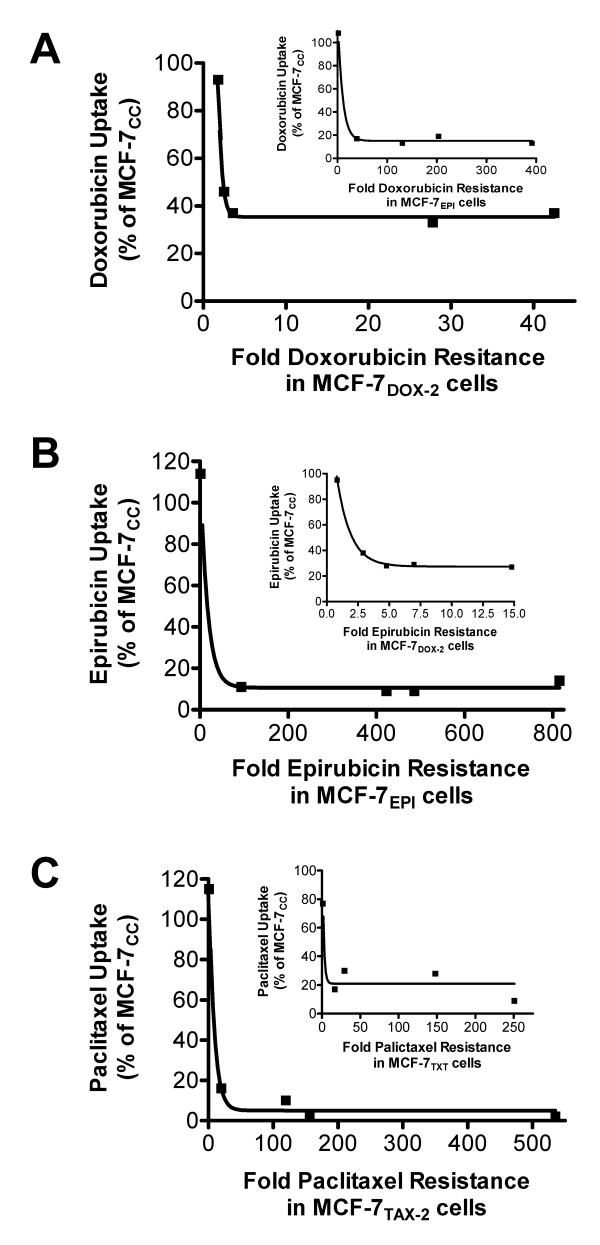
**Relationship between drug uptake and drug resistance in various drug-resistant cell lines.** Sensitivity of MCF-7_TAX-2_, MCF-7_TXT_, MCF-7_DOX-2_, and MCF-7_EPI _cells (at various selection doses) to doxorubicin, epirubicin, or paclitaxel was measured using a clonogenic assay and compared to MCF-7_CC _cells to determine fold drug resistance (the resistance factor). Uptake of doxorubicin, epirubicin, and paclitaxel was also measured in the drug-resistant cell lines and expressed relative to uptake into MCF-7_CC _cells. The relationships between doxorubicin (A), epirubicin (B), or paclitaxel (C) uptake and fold resistance to various drugs in the cell lines were then plotted.

Cells exposed to increasing concentrations of taxanes up to dose 7 showed no significant differences in radiolabelled paclitaxel accumulation compared to MCF-7_CC _cells (data not shown). Similarly, as shown in Table 2, when cells were selected to dose 8 drug levels, none of the cells exhibited significant drug accumulation defects (p > 0.30 at 24 hours). Coincident with the onset of taxane resistance at dose 9, paclitaxel uptake was markedly reduced in the MCF-7_TAX-2 _and MCF-7_TXT _cell lines to 16% and 30% of the uptake in MCF-7_CC _cells, respectively (p = 3.93^e-6 ^and 8.58^e-5 ^respectively). In both taxane-resistant cell lines, the uptake of paclitaxel continued to decrease, such that by dose 12, MCF-7_TAX-2 _and MCF-7_TXT _cells took up only 2% (p = 1.49^e-5^) and 9% (p = 3.17^e-5^) of the uptake in MCF-7_CC _cells (Table 2). Despite these findings, there did not appear to be a linear dose-dependent relationship between drug resistance and drug accumulation (Figure 2C). While statistically significant reductions in paclitaxel uptake did accompany the onset of paclitaxel resistance, further increases in drug resistance occurred with minimal changes in cellular paclitaxel uptake. This suggested that paclitaxel resistance in MCF-7_TAX-2 _and MCF-7_TXT _cells may not be solely related to changes in cellular paclitaxel accumulation, particularly at higher selection doses.

### Relationship between Drug Resistance and Cellular Doxorubicin and Epirubicin Uptake

The fluorescent nature of doxorubicin and epirubicin enabled us to directly measure by flow cytometry changes in cellular accumulation of these drugs during selection for doxorubicin and epirubicin resistance. There was no difference in doxorubicin or epirubicin uptake between drug-selected cells and MCF-7_CC _cells up to and including dose 7 (data not shown) and dose 8 (Table 2). Similar to the above paclitaxel uptake data, doxorubicin and epirubicin uptake was significantly reduced in MCF-7_DOX-2 _cells selected to dose 9, such that doxorubicin and epirubicin uptake was only 46% (p = 0.02) and 38% (p = 0.003) of uptake in MCF-7_CC _cells, respectively. The same trend was seen for MCF-7_EPI _cells, although the amount of doxorubicin and epirubicin uptake was significantly lower, representing 17% (p = 2.7^e-5^) and 11% (p = 6.21^e-5^) of the uptake seen in MCF-7_CC _cells, respectively. Also similar to our observations with the taxane-resistant cell lines, statistically significant reductions in doxorubicin or epirubicin uptake did accompany the onset of doxorubicin or epirubicin resistance, respectively. However, further increases in drug resistance were observed that did not appear to be correlated with changes in drug accumulation (Figure 2A and 2B). Again, this suggests that resistance to doxorubicin or epirubicin may involve additional mechanisms not related to drug uptake into cells.

### Relationship between Drug Resistance, Drug Accumulation and Expression of Drug Transporters

**Figure 3:**
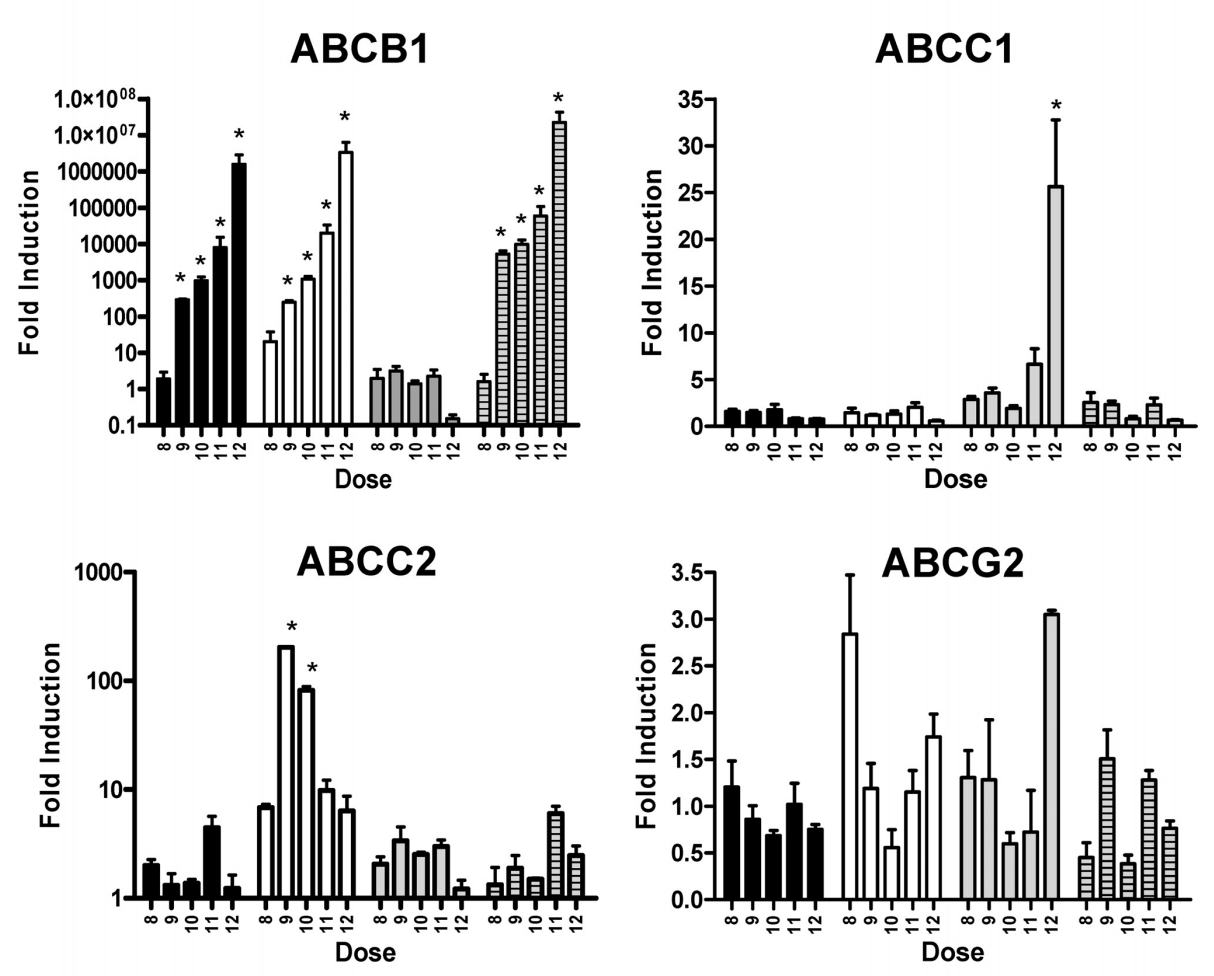
**Levels of drug transporters at various selection doses in cells selected for progressive resistance to paclitaxel, docetaxel, doxorubicin, and epirubicin (MCF-7_TAX-2_, MCF-7_TXT_, MCF-7_DOX-2_, and MCF-7_EPI _cells, respectively).** MCF-7_TAX-2_, MCF-7_TXT_, MCF-7_DOX-2_, and MCF-7_EPI _cells are represented by black, white, grey, and grey striped bars, respectively. The symbol * denotes changes in gene expression that vary significantly from levels in cells selected to dose 8 (p < 0.05).

The acquisition of drug resistance and/or changes in cellular drug accumulation observed above may be related to changes in cellular expression of drug transporters known to play a role in drug resistance. To assess this hypothesis, we used quantitative reverse transcription PCR to accurately measure the level of transcripts for the ABCB1, ABCC1, ABCC2, ABCC4, ABCG2, and LRP drug transporters. As shown in Figure 3A, acquisition of epirubicin resistance at the threshold selection dose resulted in a dramatic induction of ABCB1 gene expression [X^2^(4) = 11.067, p < 0.05]. ABCB1 transcript levels were found to increase further at higher selection doses. There also appeared to be some elevation in ABCC2 transporter expression in epirubicin resistance at higher selection doses, but such changes in gene expression were found not to be statistically significant ([X^2^(4) = 7.21, p > 0.05, ns]; Figure 3C). Elevated ABCB1 expression was also observed at the threshold dose (dose 9) in MCF-7_TAX-2 _and MCF-7_TXT_, where resistance to paclitaxel and docetaxel was first observed [X^2^(4) = 11.725, p < 0.05 and X^2^(4) = 10.495, p < 0.05, respectively]. The expression of ABCB1 increased further at higher selection doses, similar to our observations in MCF-7_EPI _cells (Figure 3A). ABCB1 appeared to be the only drug transporter which changed expression upon selection for paclitaxel resistance, whereas selection for docetaxel resistance also resulted in increased ABCC2 transporter expression ([X^2^(4) = 10.038, p < 0.05]; Figure 3C). Interestingly, selection for doxorubicin resistance did not result in detectable increases in ABCB1 expression (Figure 3A). A strong increase in ABCC1 expression (Figure 3B) was observed for MCF-7_DOX-2 _cells selected to dose 12 [X^2^(4) = 12.367, p < 0.05]. ABCG2 (Figure 3D), ABCC4 (data not shown) and LRP (data not shown) transcript levels were unchanged during selection for resistance to any of the anthracyclines or taxanes.

**Figure 4:**
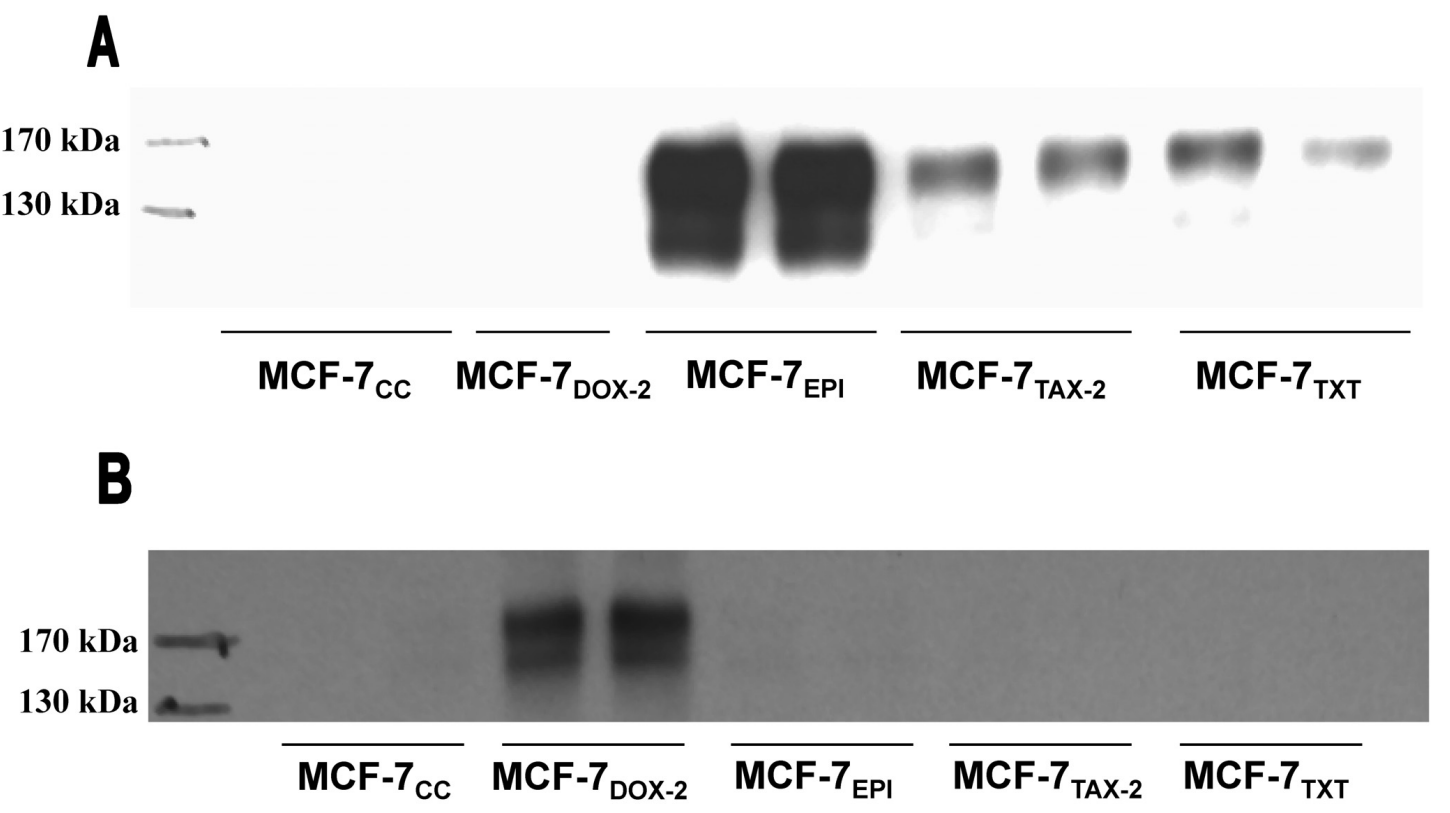
**Levels of expression of the ABCB1 and ABCC1 drug transporters in MCF-7_CC_, MCF-7_DOX-2_, MCF-7_EPI_, MCF-7_TAX-2 _and MCF-7_TXT _cell lines at selection dose 12.** The ABCB1 and ABCC1 drug transporters were immunoprecipitated from whole cell extracts using protein A/G sepharose and antibodies specific for ABCB1 (A) or ABCC1 (B). The level of expression of the drug transporters was then assessed in immunoblotting experiments using the same antibodies.

Cellular expression levels for the two most highly expressed drug transporters were also assessed at the protein level through immunoprecipitation and immunoblotting experiments using antibodies specific for ABCB1 and ABCC1. The antibodies were used both for immunoprecipitation of the drug transporters and for their quantification in subsequent Western blotting experiments. These experiments (Figure 4) demonstrated very clear evidence of a dramatic increase in ABCB1 expression in the MCF-7_EPI_, MCF-7_TAX-2_, and MCF-7_TXT _cell lines but not in the MCF-7_DOX-2_cell line. Expression was clearly much higher in MCF-7_EPI _cells than in MCF-7_TAX-2 _and MCF-7_TXT _cells). Similar experiments with an ABCC1-specific antibody revealed that ABCC1 expression was induced in MCF-7_DOX-2 _cells (selected to dose level 12), but not in any of the other drug-resistant cell lines described in our study. Our findings from immunoprecipitation/immunoblotting experiments are thus highly consistent with the levels of ABCB1 and ABCC1 mRNAs determined from quantitative PCR experiments (Figure 3).

### Effect of Cyclosporin A on Paclitaxel, Doxorubicin, and Epirubicin Uptake into Cells

**Figure 5:**
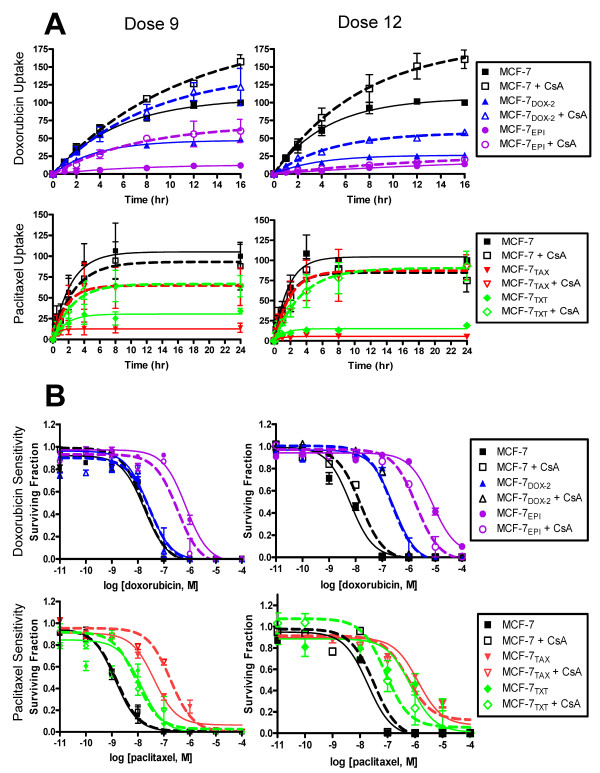
**Effects of cyclosporin A on drug uptake and drug sensitivity in co-cultured control and drug-resistant cells. **Cells selected to dose 9 or dose 12 levels of doxorubicin, epirubicin, paclitaxel, or docetaxel were pre-treated with or without 5 μM cyclosporin A and monitored for uptake of paclitaxel or doxorubicin relative to MCF-7_CC _cells (panel A). Identical cells pre-treated with or without 5 μM cyclosporin A were also assessed for their sensitivity to doxorubicin or paclitaxel using a clonogenic assay (panel B).

The above data strongly suggests that drug accumulation defects accompany the acquisition of resistance to anthracyclines and taxanes in MCF-7 cells and that this acquisition is temporally correlated with the increased expression of specific ABC transporters. However, as further drug resistance was achieved above the threshold selection dose, the degree of resistance did not highly correlate with further reductions in drug accumulation, suggesting that drug resistance may involve additional mechanisms. To address these issues, cells were pre-treated with the pan ABC drug transporter inhibitor cyclosporin A, after which drug accumulation into cells was monitored. As shown in Figure 5A, when MCF-7_DOX-2 _and MCF-7_EPI _cells (selected to dose 9) were treated with the ABC inhibitor, doxorubicin uptake into MCF-7_DOX-2 _cells was restored to levels seen in co-cultured MCF-7 cells. Doxorubicin uptake into MCF-7_EPI _cells was partially restored from 12 to 60% of uptake into MCF-7_CC _cells. Nevertheless, even in the presence of cyclosporin A, statistically significant differences in drug accumulation between the two cell lines were observed (p = 0.03). When cells selected to dose 12 were examined for doxorubicin uptake in the absence or presence of cyclosporin A, MCF-7_DOX-2 _and MCF-7_EPI _cells exhibited only a partial restoration of drug accumulation (from 26 to 58% and from 14 to 20% of uptake into MCF-7_CC _cells, respectively). Statistically significant differences in drug accumulation between MCF-7_DOX-2 _cells or MCF-7_EPI _cells and MCF-7_CC _cells were again evident in the presence of cyclosporin A (p values of 0.01 and 0.003, respectively).

Treatment of MCF-7_TAX-2 _and MCF-7_TXT _cells (selected to dose 9) with 5 μM cyclosporin A also caused a partial restoration of paclitaxel uptake in these cells (from 14 to 63% and from 34 to 64% of uptake in MCF-7_CC _cells, respectively). However, paclitaxel uptake into MCF-7_TAX-2 _cells was still found to be statistically significant from co-cultured MCF-7 cells in the presence of this agent (p = 0.006). Interestingly, when MCF-7_TAX-2 _and MCF-7_TXT _cells selected to dose 12 were treated with cyclosporin A, a complete restoration of paclitaxel uptake was observed, such that there were no differences in paclitaxel uptake between MCF-7_TXT _or MCF-7_TAX-2 _cells and MCF-7_CC _cells (p values of 0.12 and 0.23, respectively).

### Effect of Cyclosporin A on Cellular Sensitivity to Paclitaxel and Doxorubicin

While the addition of 5 μM cyclosporin A completely or partially restored uptake of doxorubicin into MCF-7_DOX-2 _cells selected to dose 9 and dose 12, respectively, this treatment exhibited little to no change in the sensitivity of cells to doxorubicin at either selection dose (Figure 4B). Treatment with cyclosporin A induced a minor 2- and 4-fold decrease in the IC_50 _for doxorubicin in MCF-7_EPI _cells selected to dose 9 and 12, respectively, suggesting a small, partial restoration of drug sensitivity. This was despite the ability of cyclosporin A to induce a 6-fold increase in doxorubicin uptake into MCF-7_EPI _cells selected to dose 9 and virtually no change in drug uptake into MCF-7_EPI _cells selected to dose 12 (Figure 4A). This suggests a clear discordance between the degree of drug resistance and the degree of drug accumulation into these drug-resistant cells. Underscoring this view, cyclosporin A induced full restoration of paclitaxel uptake into MCF-7_TAX-2 _cells selected to dose 12 but had little effect on cellular sensitivity to paclitaxel. MCF-7_TXT _cells selected to dose 12 also showed a full restoration of paclitaxel uptake in response to cyclosporin A and a substantial (8-fold) but incomplete restoration in sensitivity to paclitaxel. In MCF-7_TXT _cells selected to dose 9, the significant restoration in paclitaxel uptake by cyclosporin A did not result in any change in resistance to paclitaxel.

## Discussion

### A Threshold for Acquisition of *In Vitro *Taxane and Anthracycline Resistance at Clinically Relevant Concentrations

As described above, a number of proteins have been implicated in the ability of tumour cells to acquire resistance to chemotherapy agents. Drug transporters are highly expressed in a variety of drug-resistant cell lines, but it is unclear whether their enhanced expression is temporally or causally correlated with the acquisition of drug resistance. Even if there is a temporal relationship between transporter expression and the induction of drug resistance, it is also not known whether additional mechanisms are temporally correlated with the acquisition of drug resistance and/or whether drug transporters/cellular drug accumulation defects represent the predominant mechanism for drug resistance. It is also unknown whether acquisition of *in vitro *drug resistance takes place at clinically relevant doses. To our knowledge, this is the first study to address these issues. The data presented in this study (Table 2) strongly suggests that in selection for resistance to four different chemotherapy drugs, a specific threshold concentration of drug is required for drug resistance to be achieved. This threshold equates to "dose 9" or 3.7 nM paclitaxel, 3.3 nM docetaxel, 29 nM doxorubicin, and 31.5 nM epirubicin. At dose 9, cells acquire resistance not only to their selection agent, but also cross-resistance to an agent of similar drug class (Table 2), and in some instances to drugs of other classes (data not shown). The threshold concentration required for selection of drug resistance appears to be approximately 2-fold above the IC_50 _value for the selection agent in wild-type cells. Interestingly, this concentration is also significantly lower than that observed in the plasma of cancer patients treated with these agents [39-42], suggesting that selection for drug-resistant variants may also take place at drug doses administered to patients.

### Dose-specific Induction of Various Drug Transporters At or Above the Threshold Concentration Required for the Acquisition of Drug Resistance

To our knowledge, this is the first study to profile changes in the expression of drug transporters as cells acquire resistance during selection for survival in increasing concentrations of anthracyclines and taxanes. Through these experiments, we were able to verify that the onset of anthracycline or taxane resistance in breast tumour cells is, in some but not all instances, temporally correlated with changes in the expression of specific drug transporters (Figures 3 and 4). The transporters changing expression upon acquisition of drug resistance appear to be dependent upon the selection agent. For example, selection for resistance to paclitaxel and epirubicin resulted in a dose-dependent increase in the expression of the ABCB1 drug transporter (P-glycoprotein), without a significant change in the expression of any other drug transporter. In contrast, acquisition of resistance to docetaxel correlated with the induction of both the ABCB1 and ABCC2 transporters at dose 9. ABCB1 expression continued to increase with increasing selection dose, while ABCC2 expression fell in a dose-dependent manner after induction at dose 9. During selection with doxorubicin, the onset of doxorubicin resistance was not accompanied by any change in the expression of drug transporters associated with drug resistance. Only at the highest selection dose (dose 12) was the expression of a drug transporter induced, namely ABCC1. It appears that some other protein or mechanism was responsible for the doxorubicin accumulation defect and doxorubicin resistance observed at lower selection doses. Taken together, the data suggests that drug resistance may stem in some instances, from the combined expression of a variety of drug transporters and that the expression of drug transporters can vary with selection dose. The data also suggests that doxorubicin resistance and doxorubicin accumulation defects can occur in cells without changes in the expression of any of the known drug transporters.

### Lack of Relationship Between Drug Uptake and Drug Resistance at Low and High Selection Doses

Data from this study also illustrates an additional interesting trend. While the onset of drug resistance could be temporally correlated with reductions in drug accumulation and in some instances, changes in the expression of drug transporters, there appeared to be little correlation between the magnitude of drug resistance and reductions in drug uptake at higher selection doses (Figure 2). This suggests that additional mechanisms must be involved in the acquisition of drug resistance, particularly at higher drug concentrations. It is also possible that even at the threshold selection dose additional mechanisms unrelated to drug transporters may play a role in the observed drug resistance and drug accumulation defects. To help address these issues, we employed the use of the pan drug transporter inhibitor cyclosporin A. Although one study claimed cyclosporin A was not an effective inhibitor or substrate of the ABCG2 transporter [43], another showed that cyclosporin A could effectively inhibit the activity of the ABCB1, ABCC1, ABCG2 and LRP [44] drug transporters. Upon addition of cyclosporin A to MCF-7_DOX-2_, MCF-7_TAX-2_, MCF-7_EPI_, and MCF-7_TXT _cells selected to dose 9 or dose 12, there were significant reversals in both doxorubicin and paclitaxel accumulation defects in the cell lines (Figure 5A). At dose 9, all of the cell lines exhibited significant restorations in either doxorubicin or paclitaxel uptake, particularly for doxorubicin uptake into MCF-7_DOX-2 _cells. For cells selected to dose 12, significant restoration of doxorubicin accumulation was noted in MCF-7_DOX-2 _cells, and a complete restoration of paclitaxel uptake was observed in MCF-7_TAX-2 _and MCF-7_TXT _cells. Yet, many of these restorations in drug uptake were not accompanied by equivalent restorations in drug sensitivity (Figure 5B). This was particularly evident for doxorubicin uptake into MCF-7_DOX-2 _cells selected to dose 12 and for paclitaxel uptake into MCF-7_TAX-2 _cells selected to dose 12. These findings strongly suggest that resistance to doxorubicin and to paclitaxel cannot be attributed solely to the expression of drug transporters and/or reductions in cellular drug accumulation. Moreover, the cyclosporin A experiments further suggest that additional drug resistance mechanisms must be present in our panel of drug-resistant cell lines. Some likely additional mechanisms are described below.

While we have reported that 5 μM cyclosporin A cannot completely restore drug uptake into the drug-resistant cell lines used in this study, this appears to be in contrast to several previously published studies using cyclosporin A at concentrations ranging from 0.5 to 10 μM [16,43,45-47]. One possible explanation for this could be that the number and degree of expression of drug transporters may be higher in some cell lines employed in this study, particularly at higher selection doses. The mechanisms responsible for the drug accumulation defects may also differ amongst cell lines. While it is also possible that complete restoration of drug sensitivity could have been obtained at higher cyclosporin A concentrations, it is important to note that in both MCF-7_TAX-2_, and MCF-7_TXT _cells (at selections doses 9 and 12), full restoration of drug uptake was observed. It is acknowledged, however, that cyclosporin A concentrations may have been insufficient to completely restore drug uptake into MCF-7_DOX-2_, MCF-7_EPI _cells.

As for the effects of more specific drug transporter inhibitors, we have observed that the ABCB1-specific inhibitor valspodar could restore sensitivity to paclitaxel but not doxorubicin in similarly selected MCF-7_TAX _cells. Moreover, valspodar was unable to restore sensitivity to doxorubicin or paclitaxel in previously selected MCF-7_DOX _cells, which strongly express the ABCB1 drug transporter. Higher concentrations of valspodar had no further effect on drug sensitivity [35]. These observations suggest that inhibitors with strong affinity and specificity for ABCB1 cannot fully restore sensitivity to paclitaxel- or doxorubicin-resistant breast tumour cells. Valspodar treatment also had no effect on the localization of epirubicin in MCF-7_EPI _cells. While epirubicin is localized to the nucleus in wildtype MCF-7 cells, the drug is found in lysosomes of MCF-7_EPI _cells, suggesting that it cannot associate with its target (DNA) within the nucleus (Eng et al., manuscript in preparation). The inability of valspodar to restore drug localization to the nucleus provides further evidence that other proteins play a role in acquired resistance to anthracyclines and taxanes in breast tumour cells.

It is possible that ABCB1 gene silencing (siRNA) approaches would be more successful than cyclosporin A or valspodar in restoring drug sensitivity to drug-resistant cell lines. However, since cDNA microarray studies suggest that multiple proteins play a role in acquired drug resistance, it would be unexpected that ABCB1 siRNAs could fully restore drug sensitivity in our drug-resistant breast tumour cell lines. Recent studies also suggest that ABCB1 RNA interference approaches have mixed success in restoring drug sensitivity to drug-resistant cell lines. While ABCB1 siRNAs were able to restore drug sensitivity in daunorubicin-resistant gastric, hepatic, and pancreatic tumour cell lines [48,49], they showed little ability to restore drug sensitivity in paclitaxel-resistant PC-3-TxR prostate cancer cells [50]. The above findings thus support the hypothesis that multiple mechanisms may be involved in the acquisition of drug resistance in tumour cells.

### Additional Mechanisms of Drug Resistance At or Above the Threshold Selection Dose

What additional mechanisms could be involved at the onset or at high levels of drug resistance? cDNA microarray analysis was used recently by our research group to identify changes in gene expression that take place during selection for resistance to specific taxanes or anthracyclines. Interestingly, in addition to the drug transporters, a number of additional genes changed expression at or above the threshold selection dose. One such gene (a "1C" aldoketoreductase) increased its expression in MCF-7_DOX-2 _cells by almost 35-fold from dose 8 to dose 12 (Veitch et al., manuscript in submission). Moreover, addition of a specific pharmacological inhibitor of aldoketoreductase 1C2 (5-β cholanic acid) almost completely restored sensitivity to doxorubicin in MCF-7_DOX-2 _cells at dose 12 (Veitch et al., manuscript in submission). This strongly suggests a role for aldoketoreductases in anthracycline resistance, which may involve their ability to covert anthracyclines to less-toxic 13-hydroxy metabolites and/or block anthracycline-mediated DNA damage [51,52]. Assessment of the subcellular location of anthracyclines in MCF-7_DOX-2 _and MCF-7_EPI _cells by fluorescence microscopy further revealed that anthracycline resistance at or above the threshold selection dose could be temporally correlated with exclusion of anthracyclines from the nucleus and their localization to lysosomal vesicles for possible exocytosis from cells (Eng et al., manuscript in preparation). This nuclear exclusion of anthracyclines in anthracycline-resistant cells above threshold could not be restored by the addition of cyclosporin A or valspodar at concentrations able to restore drug uptake into these cells. Thus, mechanisms unrelated to cellular drug accumulation appear to be temporally and/or causally related to the acquisition of drug resistance in MCF-7 cells.

## Conclusion

In conclusion, this study provides an assessment of the temporal and causal relationship between the expression and activity of drug transporters in MCF-7 cells and the acquisition of resistance to anthracyclines and taxanes. Our data strongly suggests that while reduced drug accumulation and the induction of expression of various drug transporters is temporally correlated with the onset of drug resistance in MCF-7 breast tumour cells at clinically relevant drug concentrations, the magnitude of resistance appears to be poorly correlated with the degree of reduction in cellular drug accumulation, particularly at higher selection doses. In addition, resistance is not substantially reduced upon restoration of drug accumulation into cells, suggesting the presence of additional drug resistance mechanisms, two of which (for doxorubicin) likely involve the action of aldoketoreductases and changes in cellular drug localization. Future studies involving these cell lines should also help assess the temporal correlation between the acquisition of drug resistance and specific chromosomal amplifications or epigenetic changes implicated in the induced expression of drug resistance-related proteins. This increased knowledge of the relevance of various mechanisms to drug resistance *in vitro *should help identify better strategies for possible circumvention of drug resistance in cancer patients.

## List of Abbreviations

ABC Transporter : ATP Binding Cassette transporter; ABCB1 : ABC transporter family B1, also known as multidrug resistance protein 1 (MDR1) and P-glycoprotein; ABCC1 : ABC transporter family C1, also known as multidrug resistance-related protein 1 (MRP1); ABCC2 : ABC transporter family C2, also known as multidrug resistance-related protein 2 (MRP2); ABCG2 : ABC transporter family G2, also known as breast cancer resistance protein (BCRP) and mitoxantrone resistance protein (MXR); LRP : Lung resistance-related protein, also known as major vault protein (MVP).

## Competing interests

The authors declare that they have no competing interests.

## Authors' contributions

SH helped to perform the clonogenic assays, performed the paclitaxel uptake experiments, and drafted the manuscript.

ML performed the epirubicin and doxorubicin uptake experiments, performed the Q-PCR experiments, helped to perform the clonogenic assays, and read and approved the final manuscript. DV helped to perform the clonogenic assays, and read and approved the final manuscript. BG helped to perform the doxorubicin and epirubicin uptake experiments, and read and approved the final manuscript. ZV helped to perform the clonogenic assays, and read and approved the final manuscript. MC helped to perform the clonogenic assays, and read and approved the final manuscript. AP conceived of the study, raised funds for supporting the study, participated in its design and coordination, and made final changes to the manuscript.

## Pre-publication history

The pre-publication history for this paper can be accessed here:


